# Axial compression tests on CFRP strengthened CFS plain angle short columns

**DOI:** 10.1038/s41598-024-57943-w

**Published:** 2024-03-27

**Authors:** K. S. Vivek, Mohammad Adil Dar, M. I. Ali, M. Manohar, T. Sreedhar Babu

**Affiliations:** 1 Department of Civil Engineering, Vasireddy Venkatadri Institute of Technology, Guntur, 522508 AP India; 2 Department of Civil and Structural Engineering, The University of Sheffield, Sheffield, S10 2TN UK

**Keywords:** Engineering, Civil engineering

## Abstract

A comprehensive test program was performed to experimentally investigate the effect of CFRP strengthening on the axial strength and stability of CFS plain angle short columns subjected to monotonic axial compression. A total of 28 specimens were tested by varying the CFRP strengthening configurations for different column heights. Both uni-directional (CF_UD) and bi-directional (CF_BD) CFRP were considered. The influence of various parameters such as the type of CFRP, fiber orientation, and number of CFRP layers was investigated and discussed in detail. For single layer (ply) of CFRP, CF_UD-0° strengthening configuration resulted in maximum increase of axial capacity by 58.33% and 45.72% (in comparison to bare steel specimens), corresponding to 0.5 m and 1.0 m column lengths respectively. All the bare steel and skin-strengthened specimens failed predominantly due to torsional–flexural buckling mode. Additional layer of CFRP wrapping was found to enhance the axial capacity further and CF_UD-0°/BD was found to possess greater capacity in the case of double layer of CFRP. Adopting cardboard in-fill in addition to CF_UD-0° wrap has prevented the torsional mode of buckling and resulted in a peak increase of axial capacity by 192.55% and 240.61% corresponding to 500 mm and 100 mm long specimens, respectively.

## Introduction

Cold-formed steel (CFS) plain angles are widely used as a chord or web element in roof trusses and bracing in single-story metal buildings. The plain angle sections are simple profiles that can be fabricated and connected to other elements with ease. However, they are singly-symmetric (about the major principal axis) sections for which the shear center and centroid do not coincide, leading to torsional buckling coupled with major axis flexural buckling^1,2^. Also, the thin elements make them vulnerable to local buckling. This complex phenomenon drastically reduces the axial capacity, which necessitates appropriate strengthening to be carried out. Though strengthening by providing additional sections, using hot-rolled sections, and using light-weight or foam concrete is possible, the weight of the components and the entire building increases, which is highly undesirable in seismically active regions as greater inertia forces will be attracted during an earthquake. To ensure that the increase in weight of the components is as minimal as possible, fiber-reinforced polymer (FRP) strengthening is appropriate from a structural performance point of view, considering the high strength-weight ratio. Though the cost of FRP is high, it is to be noted that FRP prevents the rusting of metals and enhances the durability of the members, thereby eliminating the need for the application of anti-corrosion paints, which is also costly. The maintenance required during the service life of the components will also be very minimal by opting for FRP strengthening. Among the various types of FRP, carbon fiber-reinforced polymer (CFRP) has a high modulus of elasticity and is widely adopted in practice for strengthening purposes around the world.

The present study aims at experimentally investigating the effectiveness of various CFRP strengthening configurations with application to plain angle compression members, which has not been reported previously by any other researchers. It is to be noted that the response of the CFS members requires special attention in comparison to the hot-rolled members, especially when subjected to compression, due to their higher vulnerability to complex modes of buckling, which justifies the need for the present study. The research findings from the study will be beneficial in assessing the structural performance of the members provided with various CFRP strengthening configurations in terms of axial capacity, stiffness, and failure. Finally, the stated most effective strengthening configurations will guide the practicing engineers in choosing the appropriate strengthening configuration. A brief review of available literature in line with the current scope of the work is presented in the following paragraphs.

Thin-walled plain angle sections with fixed ends are prone to torsion dominant–flexural buckling, i.e., twisting and flexural-buckling about the major axis (TFB) when subjected to axial compression^1,2^. In addition, flexural buckling about the minor-axis may also occur, and this type of interactive buckling brings down the axial capacity of the compression member significantly. Numerous research studies on the axial compression testing of thin-walled plain and lipped angle columns have consistently validated this behavior^3–12^. Several studies on built-up columns composed of plain angle sections were also reported^13–16^.

Many studies have confirmed the feasibility of adopting carbon fiber-reinforced polymer (CFRP) to strengthen structural steel members for enhanced stiffness and strength^17–20^. Silvestre et al.^21^ carried out an experimental investigation to study the structural response of CFRP-strengthened thin-walled lipped-channel sections subjected to axial compression. A total of 19 specimens, including both short and long fixed-ended channel sections, were considered. It was reported that the adoption of a single CFRP layer improved the axial compressive strength by 15% and 20%, respectively, for short and long columns. Further, the addition of more CFRP layers would further enhance the stiffness and strength characteristics. The strengthening effectiveness of CFRP wrapping at distinct locations (i.e., total cross-section, web alone, web and flange alone, flange alone, and web-flange-lip) was also investigated. A numerical investigation aiming to assess the parameters over a wider range was also adopted, although discrepancies between the numerical and experimental results were noted in some cases, which were attributed to the lack of actual geometric imperfection measurements and the need for much more meticulous and complex modeling of the CFS-CFRP interaction to account for de-lamination.

A similar experimental investigation was carried out by Kalavagunta et al.^22^, resulting in new design rules being proposed. Retrofitting of columns with CFRP indicated a significant improvement in their axial compressive strength^23^. A maximum enhancement of 18% and 23% was reported when two layers of transverse CFRP layers were adopted in short columns and three layers of longitudinal CFRP layers for long columns, respectively, due to better confinement. Axial compression tests on G450 grade steel cold-formed short square hollow columns with CFRP strengthening were performed by Bambach et al.^24^. A total of twenty square column specimens with sectional slenderness ranging from 42 to 120 were tested. It was observed that the CFRP strengthening delayed the local buckling in slender sections, thereby improving their buckling and axial capacity. An experimental investigation on the flexural response of CFRP-strengthened structural steel angle sections (hot-rolled) was reported by Madhavan et al.^25^. Both stiffness and flexural strength were significantly enhanced due to the CFRP wrapping. Adopting a lateral CFRP layer (UD-90°) over the longitudinal CFRP layer (UD-0°) provided greater confinement, resulting in further enhanced performance. Closed section strengthening, i.e., providing a cardboard in-fill along with CFRP wrapping, resulted in a peak improvement in the flexural load capacity when compared to other strengthening arrangements using CFRP alone.

Selvaraj et al.^26^ performed flexural tests on structural steel channel sections (hot-rolled) to assess the effectiveness of CFRP wrapping on the performance improvements achieved. Six different strengthening configurations were studied. Skin-strengthening by providing a single layer of uni-directional (fibers along the length of the specimen) CFRP displayed a mean strength improvement of 6.14% when compared to bare steel specimens. Although insignificant, mean strength dropping by 1.15% was noted when bi-directional CFRP was adopted. Through the adoption of closed-section strengthening (cardboard in-fill and CFRP wrapping), a mean strength enhancement of 8.71% and 25.05% was achieved when double layers of uni-directional CFRP and bi-directional layer over uni-directional layer, respectively. The closed-section strengthening eliminated the lateral-torsional buckling failure of the specimen due to larger torsional rigidity, which otherwise occurred in bare steel specimens. A similar study on the flexural strengthening of hot-rolled steel channel sections considered various combinations of uni-directional and bi-directional CFRP^27^. A mean flexural strength improvement of 3.89%, 6.15%, 8.97%, 13.54%, 28.44%, and 35.74% was achieved by skin-wrapping of UDCF, BDCF, closed single wrapping (UDCF), BDCF, closed double wrapping (UDCF + BDCF), and closed triple wrapping (UDCF + UDCF + BDCF) strengthening configurations, respectively (where UDCF and BDCF stand for uni-directional and bi-directional CFRP, respectively). All the specimens were prone to lateral-torsional buckling. The CFRP strengthening was found to enhance the durability of the steel channel beams when subjected to artificially simulated rain during the testing^28^. A similar work was further extended to the flexural strengthening of unsymmetrical built-up channel sections^29,30^. Studies^31–33^ on the use of FRP boards for strengthening CFS composite built-up columns and beams were also available, which reported a significant enhancement in the structural performance in comparison to bare steel specimens. Recently, several advanced studies in the field of CFS construction were reported^34–36^.

From the literature review discussed above, it is evident that no findings on the axial behavior of CFS plain angle columns strengthened with CFRP have been reported so far and have been accordingly taken up in the current investigation. The fundamental aim of this study is to experimentally investigate the influence of skin and closed-section strengthening using CFRP on the stability and strength of equal-leg plain angle short columns. Based on the outcomes of this study, effective CFRP strengthening configurations with ease of implementation will be recommended. The role of various parameters, such as type of CFRP, fiber orientation, and number of CFRP layers, on the structural response will also be discussed.

## Material and specimen details

### CFS angle specimens

**Figure 1:**
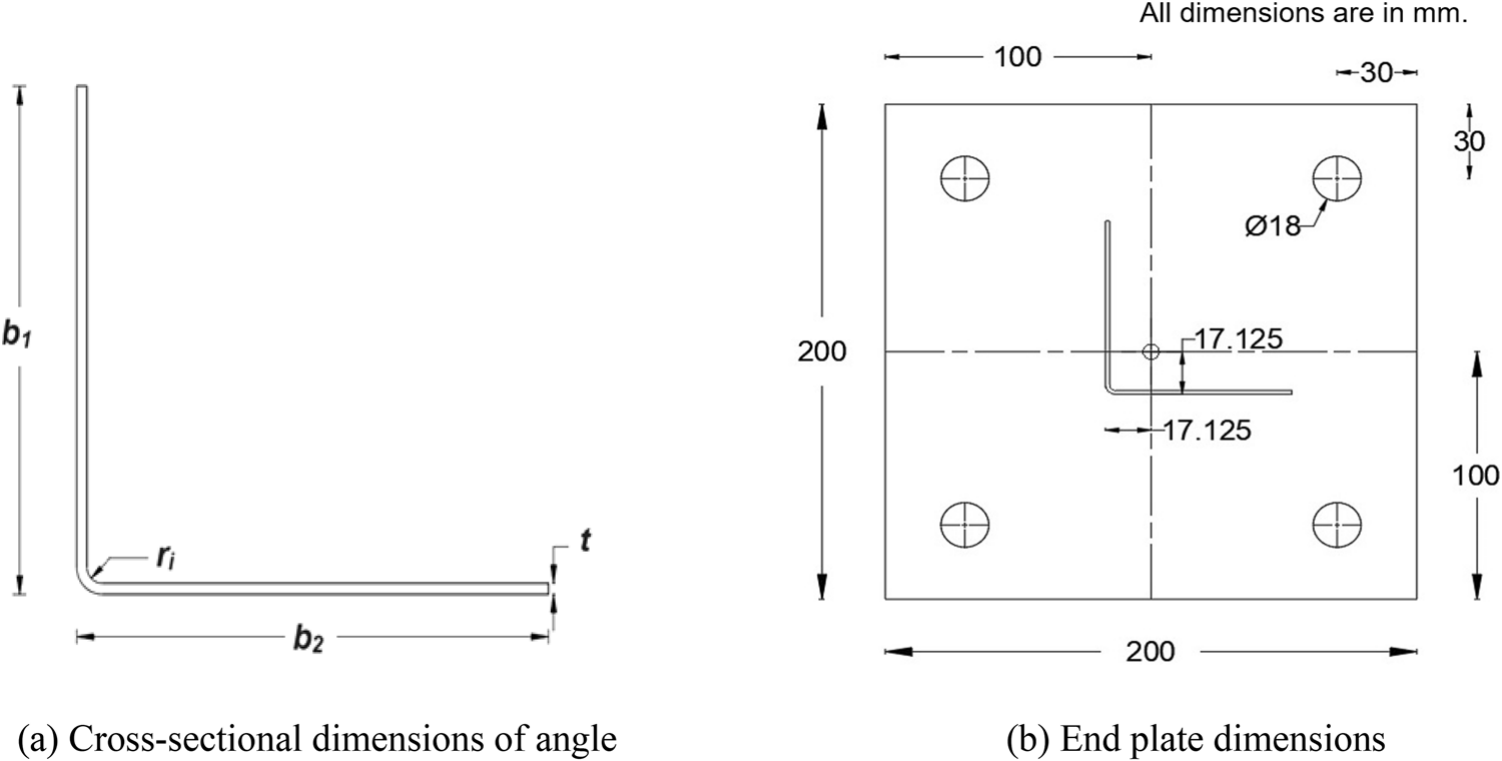
Specimen details. (**a**) Cross-sectional dimensions of angle. (**b**) End plate dimensions.

A total of 28 equal-leg plain angle specimens with nominal cross-sectional dimensions of 70 × 70 × 1.5 (all in mm) as shown in Fig. 1a were considered. Here, 16 specimens were 500 mm long (nominal length), and the remaining 12 specimens were 1000 mm long. The angle sections were formed by press-braking with the help of a Computer Numerically Controlled (CNC) press-braking machine. The actual dimensions of the angle sections were measured and are presented in Table 1. The widths of the legs were measured with the help of digital vernier calipers (accuracy of 0.01 mm), and the lengths were measured by using a measuring tape. All the angle sections were welded (at top and bottom) to square-shaped 8 mm-thick end plates of size 200 mm × 200 mm such that the centroids of both the end plates and specimen (angle section) coincide, as depicted in Fig. 1b. The end plates were provided with four bolt holes of 18 mm diameter at each corner to receive 16 mm diameter bolts.

**Table 1:** Measured dimensions and imperfections of CFS plain angle specimens.

S. no	Specimen label	b_1_ (mm)	b_2_ (mm)	t (mm)	*r*_*i*_ (mm)	L (mm)	δ_max_ (mm)
1	L500_01	69.90	69.70	1.5	2.25	495	–
2	L500_02	70.10	70.20	1.5	2.25	495	–
3	L500_03	70.13	70.00	1.5	2.25	493	–
4	L500_04	70.06	69.87	1.5	2.25	495	–
5	L500_05	69.73	69.96	1.5	2.25	497	–
6	L500_06	69.90	70.00	1.5	2.25	493	–
7	L500_07	69.96	69.93	1.5	2.25	495	–
8	L500_08	70.06	70.03	1.5	2.25	497	–
9	L500_09	70.03	70.06	1.5	2.25	495	–
10	L500_10	70.10	70.06	1.5	2.25	493	–
11	L500_11	70.03	70.13	1.5	2.25	495	–
12	L500_12	69.96	69.93	1.5	2.25	495	–
13	L500_13	70.06	70.10	1.5	2.25	495	–
14	L500_14	70.00	70.10	1.5	2.25	497	–
15	L500_15	70.06	70.00	1.5	2.25	495	–
16	L500_16	70.00	70.13	1.5	2.25	495	–
17	L1000_01	69.90	70.16	1.5	2.25	995	− 0.399
18	L1000_02	69.86	70.13	1.5	2.25	997	− 1.020
19	L1000_03	70.13	69.93	1.5	2.25	993	− 0.307
20	L1000_04	69.93	70.10	1.5	2.25	995	1.136
21	L1000_05	69.96	69.73	1.5	2.25	995	1.467
22	L1000_06	70.10	69.96	1.5	2.25	995	− 0.675
23	L1000_07	70.16	70.16	1.5	2.25	995	− 1.115
24	L1000_08	70.06	70.13	1.5	2.25	995	0.690
25	L1000_09	70.00	70.06	1.5	2.25	994	− 0.818
26	L1000_10	70.20	70.16	1.5	2.25	997	− 1.078
27	L1000_11	69.84	69.77	1.5	2.25	998	− 0.776
28	L1000_12	70.10	70.05	1.5	2.25	995	− 0.763

**Figure 2:**
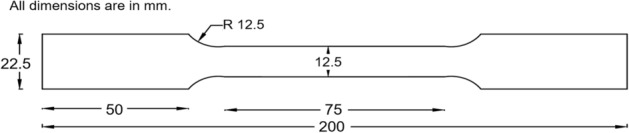
Dimensions of coupon.

**Figure 3:**
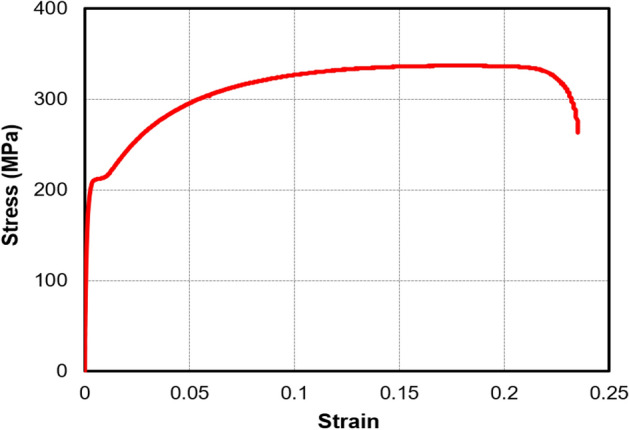
Stress–strain curve.

The mechanical properties of the CFS specimens were obtained by extracting coupons from the steel coil used for forming the angle sections. The dimensions of the flat coupons (Fig. 2) were proportioned by referring to the appropriate literature^37,38^. The tensile test was performed with the help of a computerized, servo-controlled universal testing machine (UTM) with a maximum capacity of 500 kN. An extensometer was attached to the specimen to extract accurate strain values. The averages of the modulus of elasticity (*E*), yield stress (*f*_*y*_), ultimate stress (*f*_*u*_), yield strain (ε_y_), and ultimate strain (*ε*_*u*_) were noted as 160 GPa, 208 MPa, 337 MPa, 0.002 mm/mm, and 0.18 mm/mm, respectively. The stress-strain curve obtained from the average values of stress and strain data is shown in Fig. 3.

**Figure 4:**
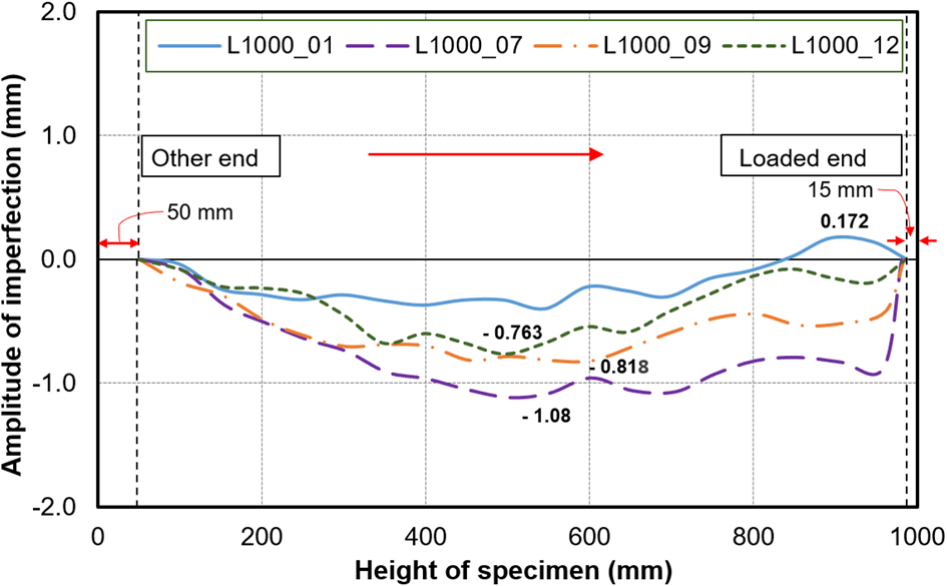
Global imperfection amplitude.

Due to low global slenderness in specimens with 0.5 m length, the initial imperfections were only recorded in 1.0 m long specimens measured with the help of a digital dial gauge (with a measuring range of 0–12.7 mm and an accuracy of 0.001 mm). A similar technique has been used previously^5^. The recorded peak global imperfection ‘*δ*_*max*_’ values were presented in Table 2. The graphical representation of the variation of initial global imperfection along the length of the selected specimens is depicted in Fig. 4.

**Table 2:** Properties of carbon fiber fabric furnished by the manufacturer.

Physical/mechanical property	Uni-directional (UD)	Bi-directional (BD)
Aerial weight (gsm)	230	200
Tensile modulus (GPa)	243	238
Tensile strength (MPa)	4081	3530
Thickness, t_CF_ (mm)	0.13	0.22
Elongation (%)	≥ 1.7	≥ 1.3

### CFRP and strengthening configurations

Both uni-directional (UD) and bi-directional (BD) carbon fiber (CF) plain-woven fabrics were procured. The mechanical properties of the fabric furnished by the manufacturer are presented in Table 2. An epoxy resin and a hardener available by the commercial names ARALDITE–LY556 and ARADUR–HY9, both manufactured by Huntsman, were used. The important properties of the resin and hardener are presented in Table 3. The resin-to-hardener mix ratio of 10:1 (by weight) was adopted as per the Huntsman technical data sheet, which is suggested for structural strengthening applications.

**Table 3:** Mechanical properties of resin and hardener.

Mechanical property	Specification
Viscosity of ARALDITE–LY556 (@25°c)	10,000–12,000 mPa s
Viscosity of ARADUR–HY951 (@ 25°c)	10–20 mPa s
Density of ARALDITE–LY556 (@25°c)	1.15–1.20 g/cc
Density of ARADUR–HY951 (@ 25°c)	0.97–0.99 g/cc
Mix ratio (epoxy resin:hardener by weight)	10:1
Viscosity of resin-hardner mix	
@ 25°c	1700 mPa s
@ 40°c	650 mPa s
Gel time	
@ 25°c	120–180 min
@ 40°c	30 min
Tensile strength of resin-hardener mix (kg/mm^2^)	6–7

**Figure 5:**
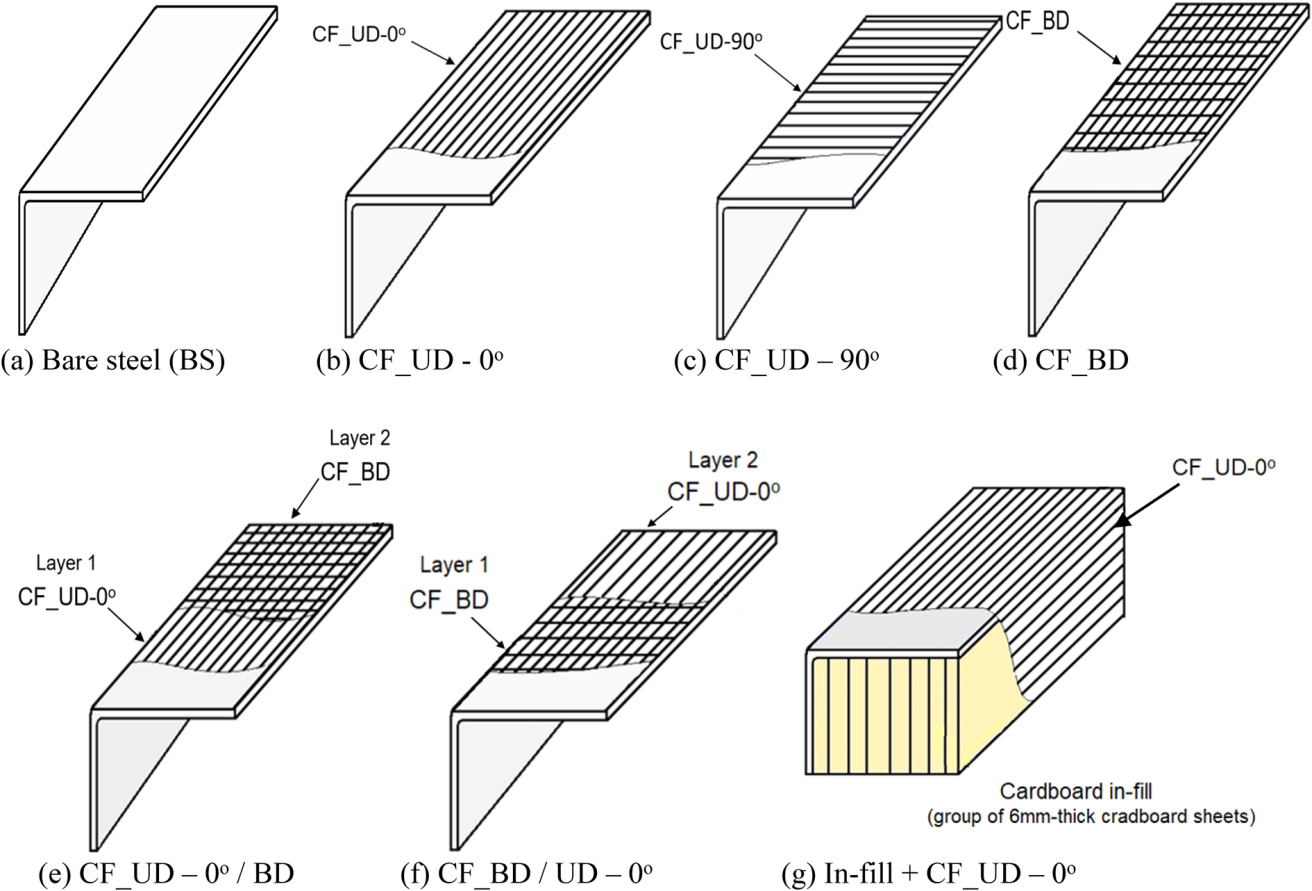
Schematic view of bare steel and CFRP strengthened angle specimens. (**a**) Bare steel (BS). (**b**) CF_UD-0°. (**c**) CF_UD-90°. (**d**) CF_BD. (**e**) CF_UD-0°/BD. (**f**) CF_BD/UD-0°. (**g**) In-fill + CF_UD-0°.

In addition to bare steel specimens (BS) as depicted in Fig. 5a, six different CFRP wrapping configurations for strengthening (as depicted in Fig. 5b–g) were considered. Out of these six, three were single-layered skin-strengthening (SLSS) configurations designated as ‘CF_UD-0°’, 'CF_UD-90o', and ‘CF_BD’ (see Fig. 5b–d, respectively). In the case of UD, 0° and 90° in designations represent carbon fibers oriented parallel and perpendicular to the length of specimens, respectively. Out of the remaining three configurations, two were double-layered skin-strengthening designated as ‘CF_UD-0°/BD’ and ‘CF_BD/UD-0°’ (see Fig. 5e,f, respectively), in which the terms on either side of ‘/’ represent the different layers of CFRP. The last one was a closed-section single-layered CFRP strengthening (CSSLS) configuration, i.e., a combination of cardboard in-fill and single-layered CFRP wrapping, designated as ‘In-fill + CF_UD-0°’ (see Fig. 5g).

All the test specimens were cleaned well on both sides (inner and outer surfaces) to remove the surface dirt present before the wrapping of CFRP (SLSS and CSSLS specimens). The outer faces of the angle specimens (which were to be strengthened with CFRP) were roughened thoroughly with the help of sandpaper to ensure proper bonding between the epoxy and the metal. For the SLSS specimens (CF_UD-0°, CF_UD-90°, and CF_BD), first the required type, size, and number of CF fabric pieces were cut and kept ready for adoption. Then, the required quantity of resin-hardener mix (10:1 proportion by weight) was prepared (thoroughly mixed with no lumps) in a plastic bowl, i.e., approximately 15–25 g of ARALDITE-LY556 epoxy resin and correspondingly 1.5–2.5 g of ARADUR-HY951 hardener for a single angle specimen. Then, using a paintbrush, the resin-hardener mix was applied uniformly to the outer faces of the angle specimens, and immediately the corresponding CF fabric was wrapped on the external faces of the specimen, followed by multiple times of pressing with the help of a roller to prevent the formation of air bubbles^21–26^. This entire process of preparing the mix and attaching the CFRP to the angles was completed within 15–25 minutes for preparing two–three specimens in a single round. After about 30-45 minutes, a fresh mix was prepared again and was applied on top of the glued CF fabric to form a CFRP lamina*,* upon which the specimens were left undisturbed for air curing over a period of 1 week prior to testing^21–26^.

In DLSS specimens (CF_UD-0°/BD and CF_BD/UD-0°), the process of attaching the first layer (Layer 1/inner layer) of CFRP was the same as discussed previously for SLSS. However, the second layer (Layer 2/outer layer) of CF fabric is also glued at the time of applying the resin-hardener mix on top of the first layer of CF. The rest of the process is the same, as discussed earlier. To fabricate CSSLS specimens (Fig. 5g), 6 mm-thick cardboard sheets were cut into the required dimensions (70 mm × 500 mm and 70 mm × 1000 mm). Based on the length of the specimens, these cardboard sheets were cut and joined together by applying a timber adhesive (commercially named FEVICOL) along the adjoining surfaces. It was then left to dry for 24 hours to form a solid in-fill. Thereafter, the extra projections were removed to form smooth edges. A special adhesive (supplied by the cardboard suppliers for attaching cardboard to the metal surfaces) was applied to the outermost surfaces of the cardboard in-fill and the inner faces of the angle specimens, which rest against each other. Afterwards, the in-fill was gently placed between the legs of the angle sections and pressed to ensure proper contact between the two. Further, it was left undisturbed for a period of 24 hours so that a strong bond could develop. Then, a resin-hardener mix was prepared to attach the CF–UD_0° fabric to the specimen. The mix was also applied to the outer faces of the angle specimens and the exposed in-fill to form the CFRP lamina. The specimen was air dried for a week prior to testing. Also, it is worth mentioning that no overlap of the CFRP was provided in any specimen, except in ‘In-fill + CF_UD-0°’, where an overlap of 100 mm was provided.

### Test setup

**Figure 6:**
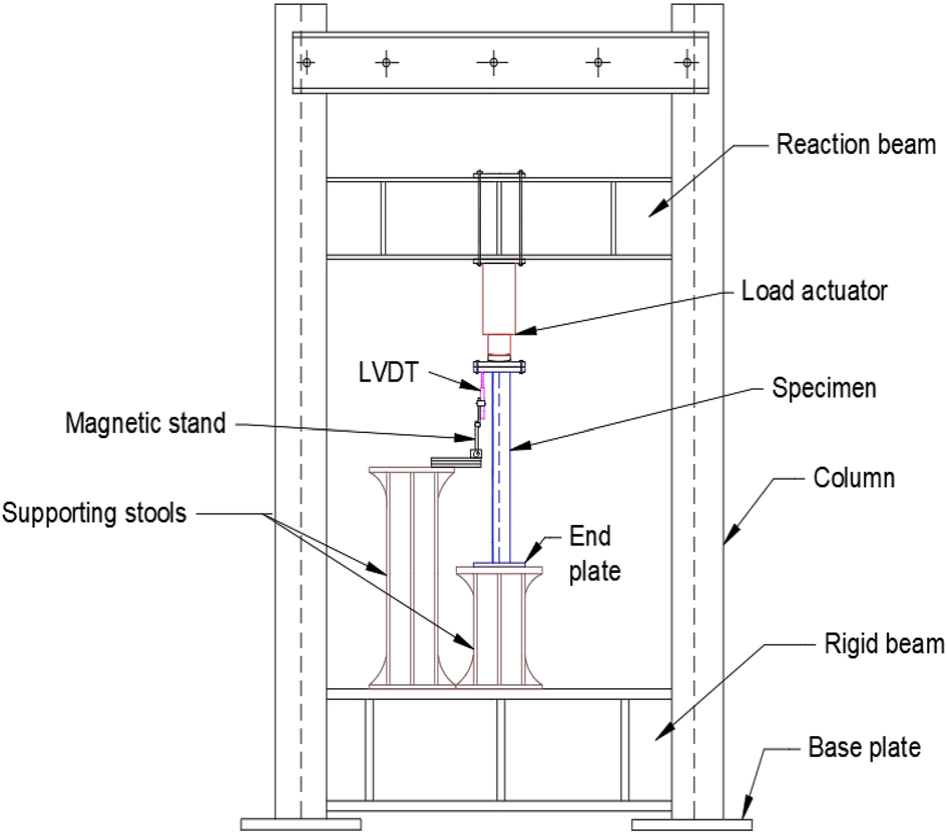
Schematic view of Loading Frame and Test Set-up.

**Figure 7:**
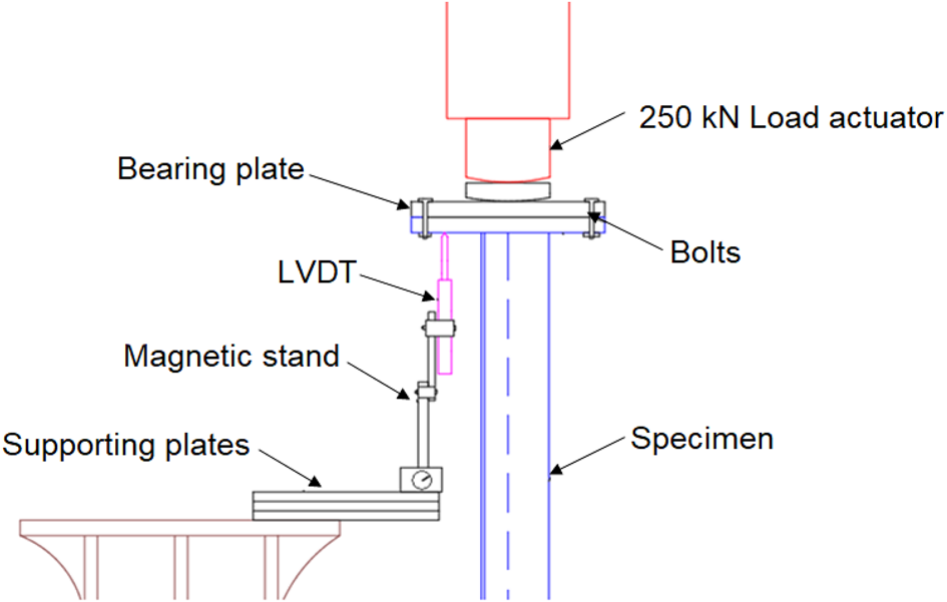
Close-up view of top end plate.

Axial compression tests were carried out with the help of a loading frame provided with 25 Ton (250 kN) hydraulic actuator (with a 150-mm stroke length), as depicted in Fig. 6. A bearing plate of 20 mm thick (200 mm wide) was provided over the top of the upper end plate (i.e., at the loaded end) to prevent localized bearing failure of the angle specimen (Fig. 7). As both the bearing plate and the upper end plate were of the same size, the centroids of the plates and the angle section coincide, as shown in Fig. 1b. Due care was taken to ensure the right contact at the marked center of the upper plate and the actuator to ensure pure axial compression, i.e., with minimal eccentricity. Further, both the plates at the top were connected to each other using four bolts of 16 mm diameter (18 mm diameter bolt holes) to prevent any possible slip. All these measures were taken properly during the loading of all the specimens. However, the top end of the specimens is free to rotate in-plane and out-of-plane, which typically represents the splice connection provided in practice. The axial displacement was recorded at regular intervals with the help of the LVDT (carrying a travel length of 50 mm). Both the actuator and LVDT were connected to a digital data acquisition system. Although LVDTs were also provided to record the lateral deformations in the specimens, the buckling of the specimens occurred at locations away from these lateral LVDTs. During the testing, the loading process was halted when the first drop in the axial capacity of the specimens was noted.

### Test results

**Figure 8:**
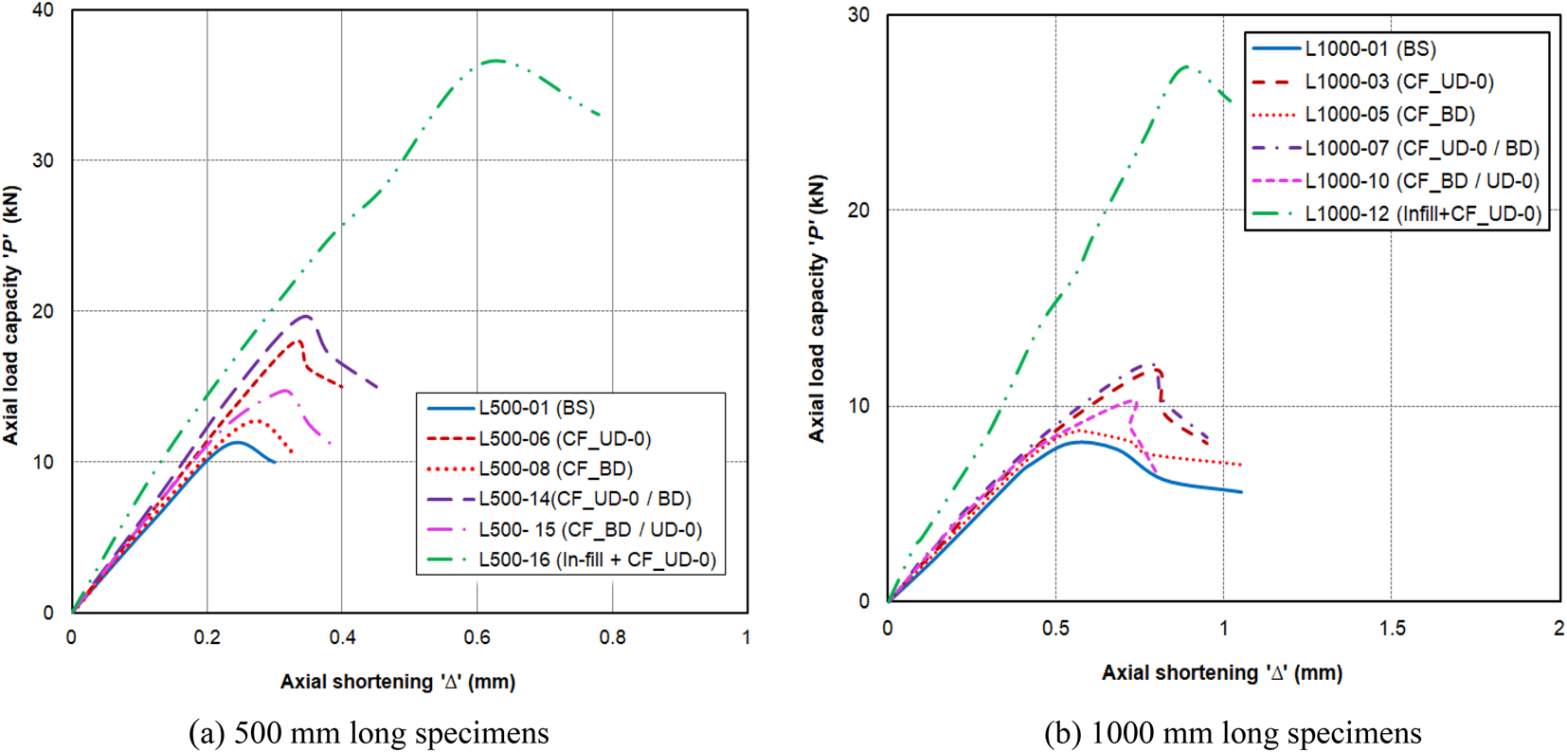
‘*P*’ vs ‘*Δ*’ plot. (**a**) 500 mm long specimens. (**b**) 1000 mm long specimens.

**Figure 9:**
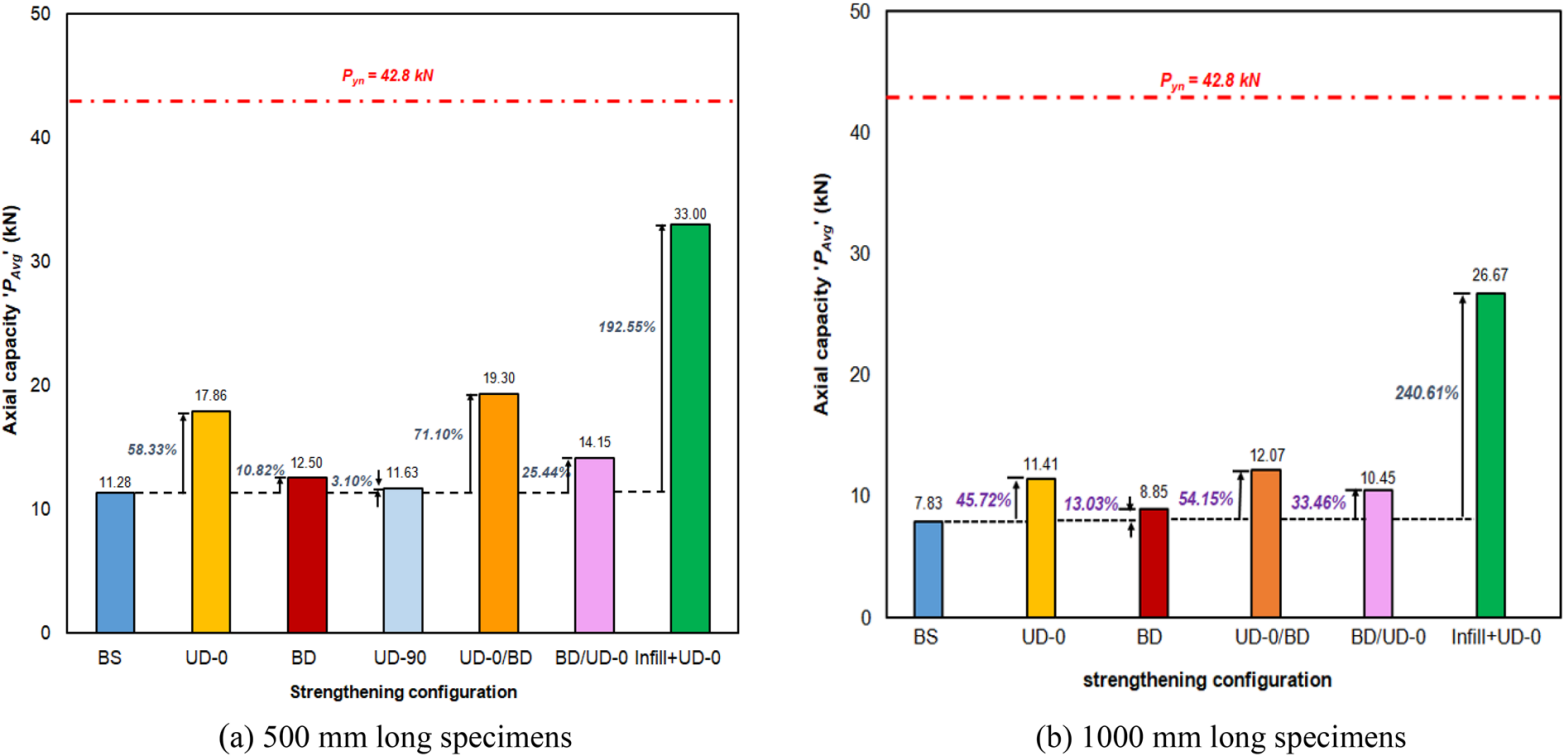
Influence of various CFRP strengthening configurations on axial capacity. (**a**) 500 mm long specimens. (**b**) 1000 mm long specimens.

**Figure 10:**
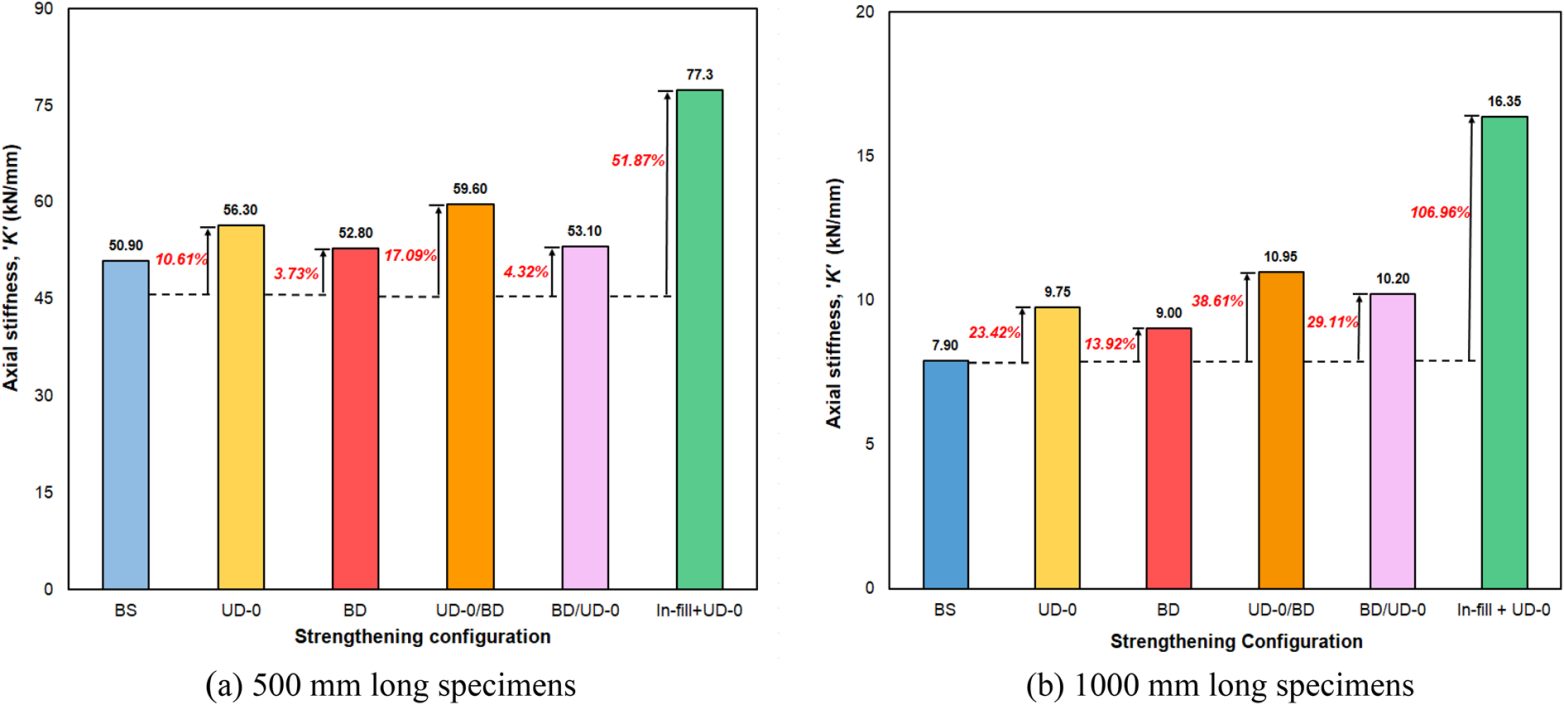
Influence of various CFRP strengthening configurations on axial stiffness. (**a**) 500 mm long specimens. (**b**) 1000 mm long specimens.

**Figure 11:**
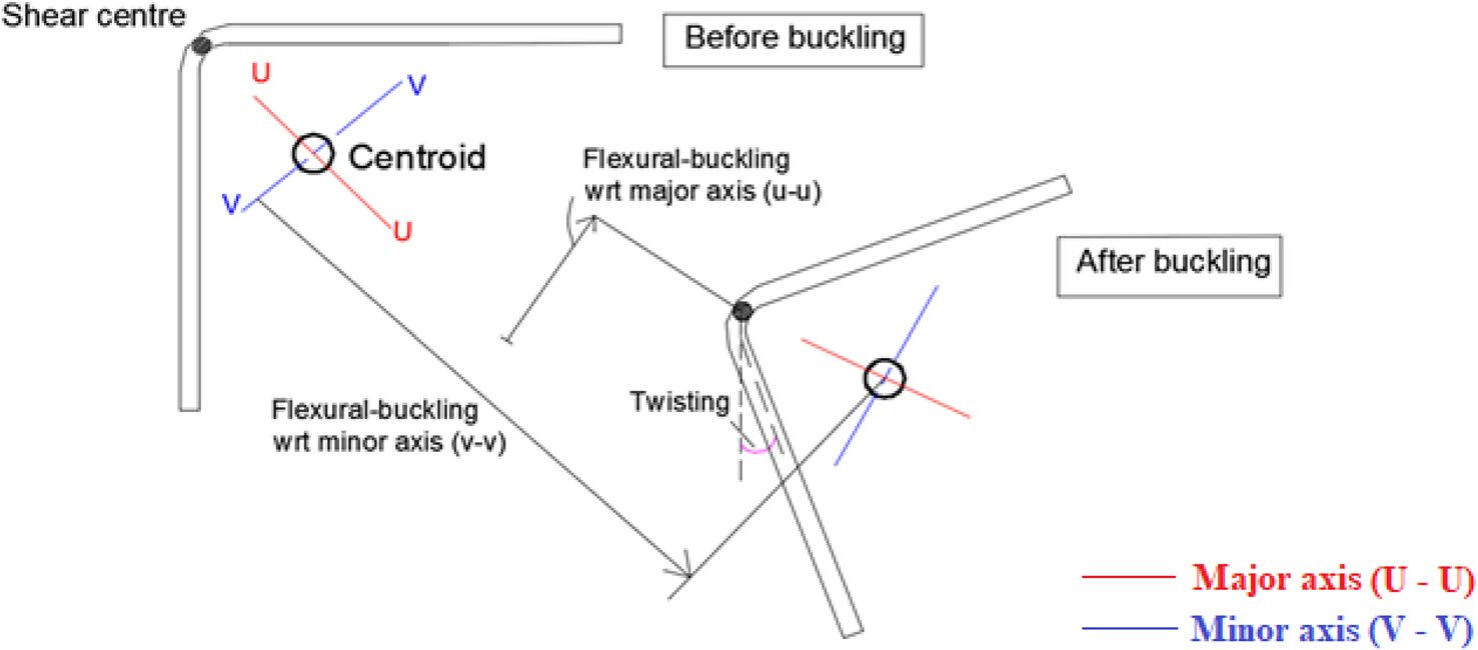
Torsional-flexural buckling (TFB).

The maximum axial load carrying capacities ‘*P*_*max*_’ and the corresponding axial shortening ‘*Δ*_*max*_’ for 500 mm and 1000 mm long specimens are presented in Tables 4 and 5, respectively. Each test was repeated to ensure the reliability, accuracy, and generalizability of the outcomes. The mean axial capacities ‘P_*max, avg*_’ and corresponding mean axial shortening ‘∆_*max,avg*_’ were also presented in Tables 4 and 5. The nominal yield strength (P_yn_) of the section was calculated to be 42.8 kN. The graphical representation of the ‘*P*’ vs. ‘*Δ*’ plots for selected specimens of 500 mm and 1000 mm lengths is depicted in Fig. 8a,b, respectively. The effectiveness of various CFRP configurations considered in the study, i.e., the percentage increase in axial strength for 500 mm and 1000 mm long specimens, is depicted in Fig. 9a,b, respectively. The same for stiffness improvements is depicted in Fig. 10a,b, respectively. All the skin-strengthened specimens were prone to torsion-dominant flexural buckling (see Fig. 11).

**Table 4:** Test results of 500 mm long specimens.

Specimen label	Strengthening scheme	*P*_*max*_ (kN)	*P*_*max, avg*_ (kN)	*P*_*max, avg*_/*P*_*yn*_	*∆*_*max*_ (mm)	*∆*_*max, avg*_ (mm)	Failure mode
L500- 01	Bare steel (BS)	11.20	11.28	0.26	0.23	0.21	LB + TFB + FB
L500-02		11.16			0.21		LB + TFB + FB
L500-03		11.50			0.20		LB + TFB** + **FB
L500-04	CF_UD-0° (SLSS)	17.66	17.86	0.42	0.29	0.31	TFB + FB
L500-05		17.95			0.33		TFB + FB
L500-06		17.97			0.31		TFB + FB
L500-07	CF_BD (SLSS)	12.73	12.50	0.34	0.28	0.26	TFB + FB
L500-08		12.27			0.24		TFB + FB
L500-09	CF_UD-90° (SLSS)	11.19	11.63	0.27	0.25	0.23	TFB + FB
L500-10		12.06			0.20		TFB + FB
L500-11	CF_UD-0°/BD (DLSS)	18.97	19.30	0.45	0.36	0.35	TFB + FB
L500-12		19.63			0.34		TFB + FB
L500-13	CF_BD/UD-0° (DLSS)	13.60	14.15	0.33	0.32	0.31	TFB + FB
L500-14		14.69			0.31		TFB + FB
L500-15	In-fill + CF_UD-0° (CSSLS)	33.00	34.75	0.81	0.73	0.67	Crushing of cardboard in-fill + rupture of CFRP
L500-16		36.50			0.61		

*LB* local buckling, *TFB* torsional–flexural (major-axis) buckling, *FB* flexural (minor-axis) buckling.

**Table 5:** Test results of 1000 mm long specimens.

Specimen label	Strengthening scheme	*P*_*max*_ (kN)	*P*_*max,avg*_ (kN)	*P*_*max,avg*_/*P*_*yn*_	*∆*_*max*_ (mm)	*∆ *_*max, avg*_ (mm)	Failure mode
L1000-01	Bare steel (BS)	8.13	7.83	0.18	0.54	0.54	TFB + FB
L1000-02		7.53			0.55		TFB + FB
L1000-03	CF_UD-0° (SLSS)	11.88	11.41	0.27	0.75	0.76	TFB + FB
L1000-04		10.94			0.77		TFB + FB
L1000-05	CF_BD (SLSS)	8.78	8.85	0.21	0.56	0.56	TFB + FB
L1000-06		8.91			0.57		TFB + FB
L1000-07	CF_UD-0°/BD (DLSS)	12.19	12.07	0.28	0.79	0.78	TFB + FB
L1000-08		11.94			0.78		TFB + FB
L1000-09	CF_BD/UD-0° (DLSS)	10.59	10.45	0.24	0.76	0.74	TFB + FB
L1000-10		10.31			0.73		TFB + FB
L1000-11	In-fill + CF_UD-0° (CSSLS)	26.03	26.67	0.62	0.85	0.86	Crushing of cardboard in-fill + rupture of CFRP
L1000-12		27.31			0.88		

*TFB* torsional–flexural (major-axis) buckling, *FB* flexural (minor-axis) buckling.

### Axial capacity

In the case of 500 mm long specimens, the mean axial capacities ‘*P*_*max,avg*_’ of BS, CF_UD-0°, CF_BD, CF_UD-90°, CF_UD-0°/BD, CF_BD/UD-0°, and in-fill + CF_UD-0° labeled specimens were obtained as 11.28 kN, 17.86 kN, 12.50 kN, 11.63 kN, 19.30 kN, 14.15 kN, and 34.75 kN, respectively, corresponding to mean axial shortening (at maximum capacities) ‘∆_*max,avg*_’ 0.21 mm, 0.31 mm, 0.26 mm, 0.23 mm, 0.35 mm, and 0.31 mm. The corresponding *P*_*max*_*,*_*AVg*_*, and P*_*yn*_ ratios for the specimens were 0.26, 0.42, 0.34, 0.27, 0.45, and 0.33, respectively.

For 1000 mm long specimens, the mean axial capacities of BS, CF_UD-0o, CF_BD, CF_UD-0o/BD, CF_BD/UD-0° and in-fill + CF_UD-0° labeled specimens were obtained as 7.83 kN, 11.41 kN, 8.85 kN, 12.07 kN, 10.45 kN, and 26.67 kN, respectively, corresponding to mean axial shortening of 0.54 mm, 0.76 mm, 0.56 mm, 0.78 mm, 0.74 mm, and 0.86 mm. It is to be noted that the CF_UD-90° strengthening configuration was not adopted due to the marginal influence on axial capacity noticed in the case of 500 mm-long specimens. In the case of SLSS, CF_UD-0° resulted in a maximum increase in axial strength of 58.33% and 45.72%, respectively, for 500 mm and 1000 mm long specimens in comparison to BS specimens. Similarly, in the case of DLSS, CF_UD-0° and BD resulted in a maximum increase in axial strength of 71.10% and 54.15%, corresponding to 500 mm and 1000 mm long specimens, respectively.

It is to be noted that in the case of SLSS, CF_UD-0o was found to be most effective as the fibers were along the length of the specimens (fiber density along the direction of loading). The fibers embedded in the resin act as 'micro-columns' in resisting the applied axial load until cracking of the resin matrix occurs. CF_BD has fibers aligned in both directions, i.e., a lesser fiber density (a smaller number of fibers embedded) in the direction of loading, and hence could not outperform CF_UD-0° was the least effective strengthening configuration as the fibers were not oriented in the direction of loading (i.e., length of specimen). Similarly, in the case of DLSS, CF_UD-0°/BD outperformed CF_BD/UD-0° because the first layer of the former was CF_UD-0°, possessing greater fiber density along the direction of loading.

The use of cardboard infill resulted in an increase in axial strength of 192.55% and 240.61% for 500 mm and 1000 mm long specimens, respectively. This is due to the fact that the infill transforms the open section into a closed section, which increases the torsional rigidity of the section, i.e., prevents torsional buckling, and also directly contributes to the axial capacity.

### Axial stiffness

The initial axial stiffnesses ‘*K*’ of all the specimens were obtained from the respective ‘*P*’ vs. ‘*∆*’ plot (Fig. 8), corresponding to an axial shortening of 0.1 mm. The initial axial stiffness resembles the ability of the specimens to resist axial compression. In the case of 500 mm long specimens, the initial axial stiffnesses of BS, CF_UD-0°, CF_BD, CF_UD-0°/BD and CF_BD/UD-0° and in-fill + CF_UD-0° labeled specimens were obtained as 50.90 kN/mm, 56.30 kN/mm, 52.80 kN/mm, 59.60 kN/mm, 53.10 kN/mm, and 77.3 kN/mm in the case of 500 mm long specimens, and 7.9 kN/mm, 9.75 kN/mm, 9.00 kN/mm, 10.95 kN/mm, 10.20 kN/mm, and 16.35 kN/mm in the case of 1000 mm long specimens, respectively.

Considering SLSS, CF_UD-0° possesses greater confinement and prevents local buckling leading to much higher axial stiffness in comparison to CF_BD and CF_UD-90o. Due to the same reason, CF_UD-0°/BD outperformed CF_BD/UD-0° in the case of DLSS. For the case of CSSLS, the cardboard in-fill prevented buckling and hence resulted in peak increase of axial stiffness.

### Failure modes

**Figure 12:**
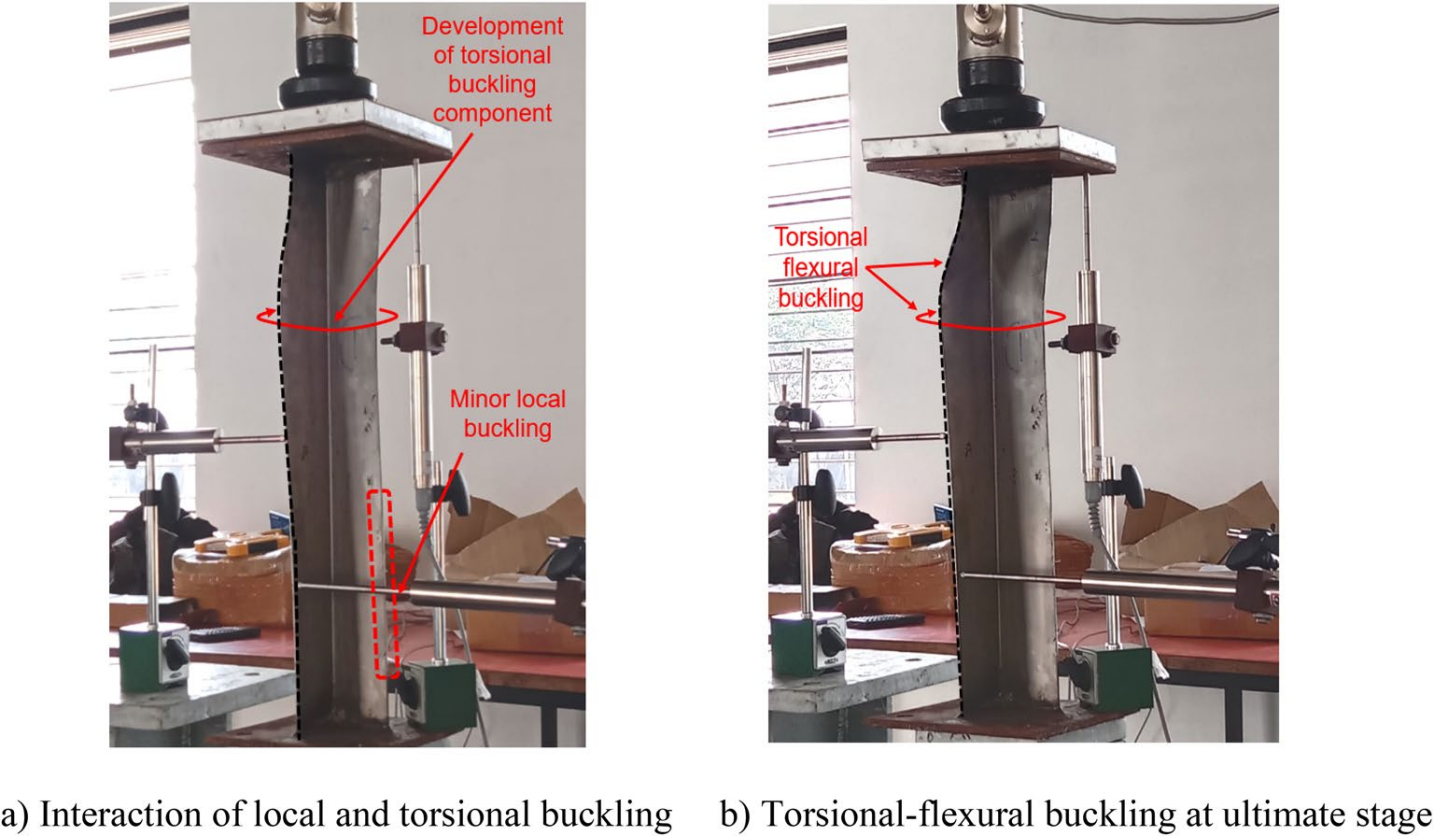
Specimen L500-01 (BS). (**a**) Interaction of local and torsional buckling. (**b**) Torsional-flexural buckling at ultimate stage.

**Figure 13:**
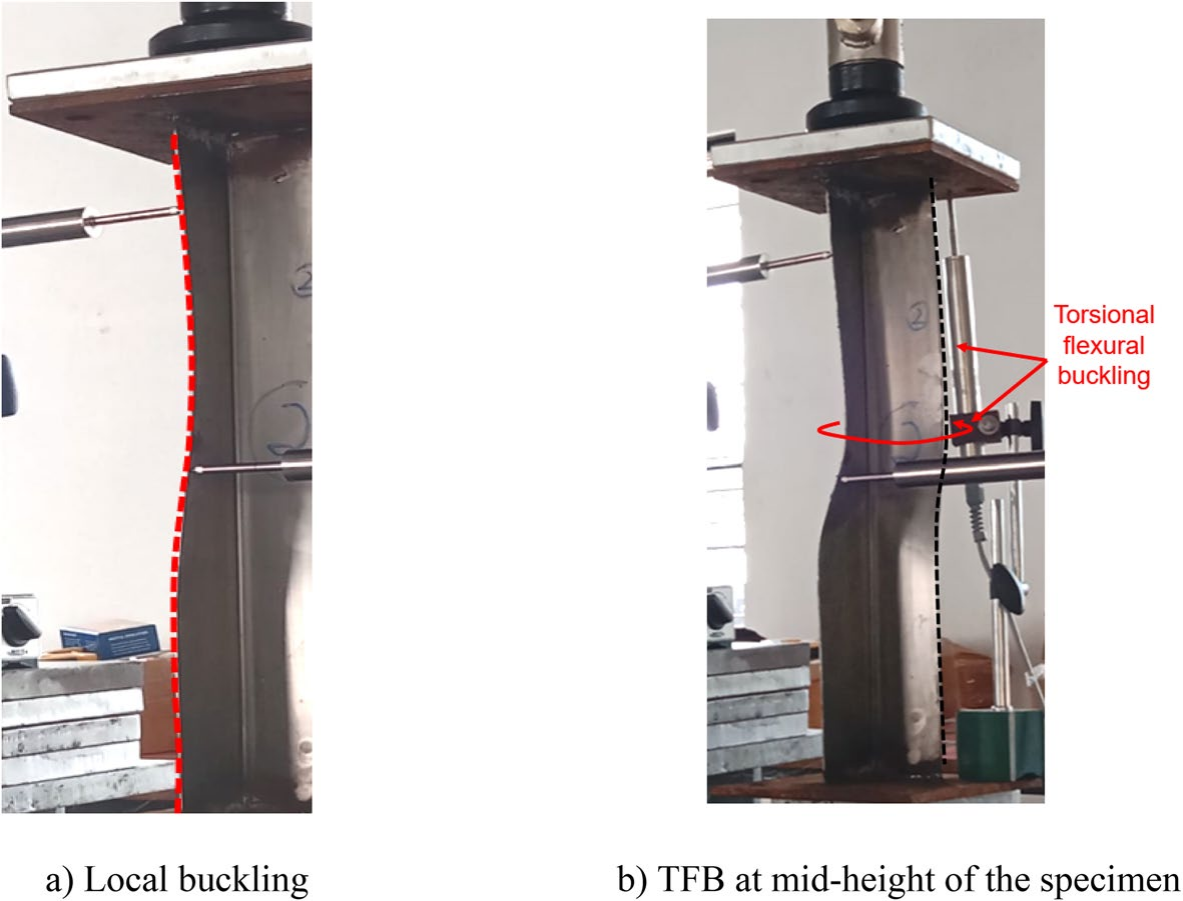
Specimen L500-02 (BS). (**a**) Local buckling. (**b**) TFB at mid-height of the specimen.

**Figure 14:**
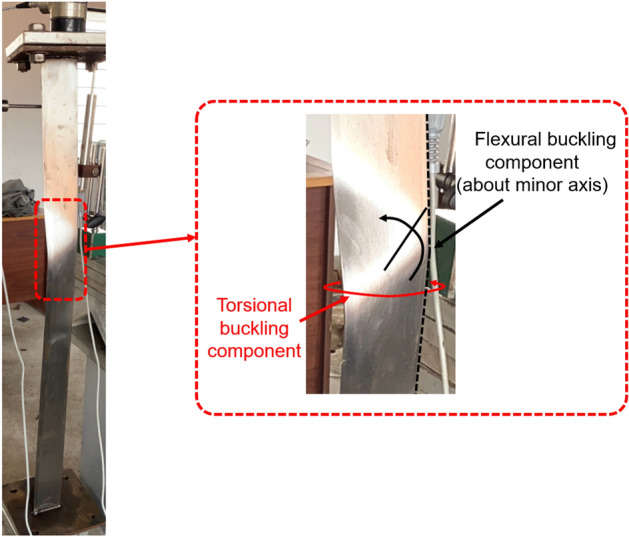
Failure of specimen L1000-01 (BS).

**Figure 15:**
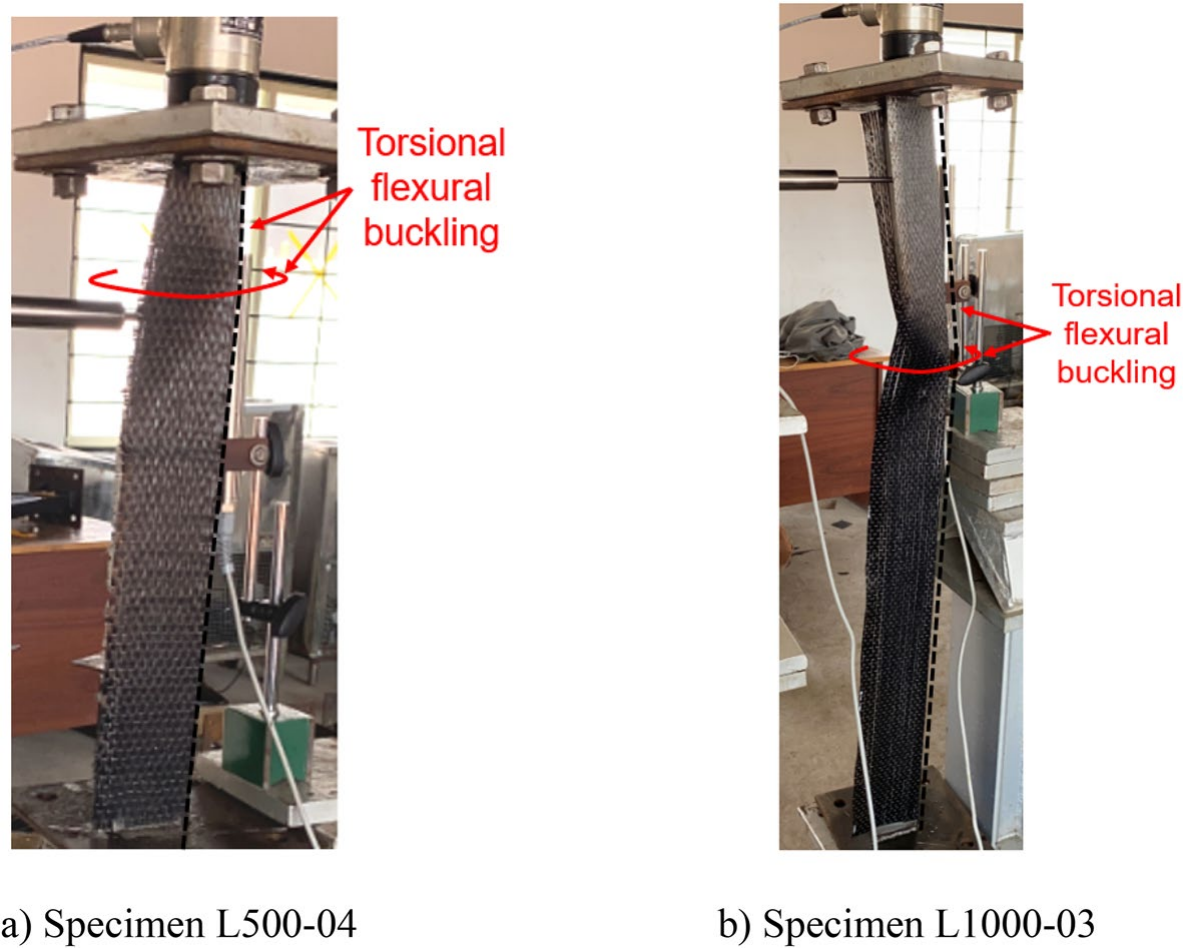
Failure of CFS_UD-0° specimens. (**a**) Specimen L500-04. (**b**) Specimen L1000-03.

**Figure 16:**
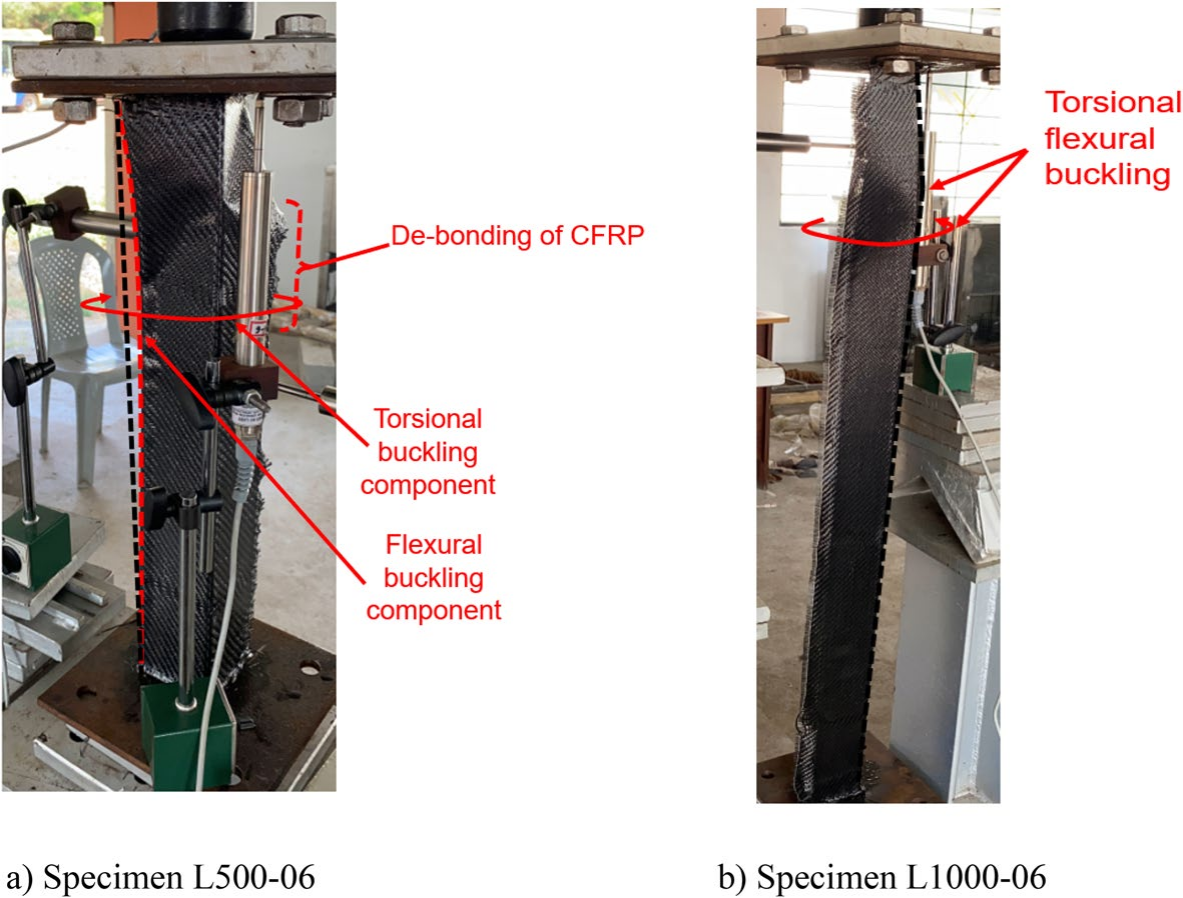
Failure of CF_BD specimens. (**a**) Specimen L500-06. (**b**) Specimen L1000-06.

**Figure 17:**
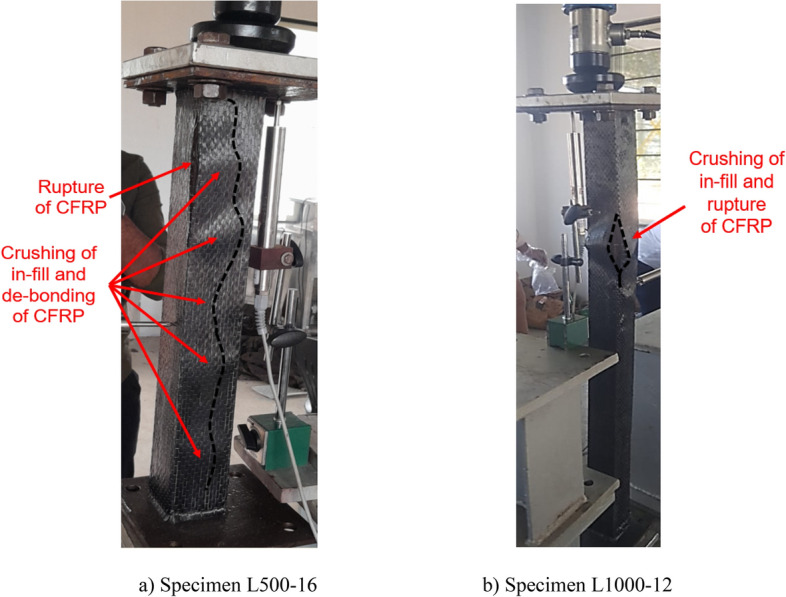
Failure of In-fill + CF_UD-0° specimens. (**a**) Specimen L500-16. (**b**) Specimen L1000-12.

All the BS specimens and CFRP-strengthened specimens without infill (i.e., SLSS and DLSS) failed by a combination of torsion dominant-flexural (major axis) buckling (TFB) and minor-axis flexural buckling (FB), as depicted in Fig. 11. In the case of 500-mm-long BS specimens, a minor local buckling (LB) deformation in one of the legs of the angle section (close to the other column end) occurred, as shown in Fig. 12a. No LB was visible in the 1000-mm long bare steel specimens. Likewise, no local buckling of CFRP-strengthened specimens was noted, irrespective of their length. The failure modes of specimens L500-01 (BS), L500-2 (BS), L1000-01 (BS), L500-04 (CF_UD-0o), L1000-03 (CF_UD-0o), L500-06 (CF_BD), and L1000-06 (CF_BD) were depicted in Figs. 12, 13, 14, 15a,b, 16a,b, respectively. De-bonding of CFRP was noticed in CFRP skin-strengthened specimens at the locations of buckling. In the case of CSSLS specimens, the failure was due to the crushing of the infill and the rupture of CFRP, as shown in Fig. 17a,b, corresponding to specimens L500-16 and L1000-12, respectively.

## Conclusions

The research findings of an experimental investigation aimed to assess the load carrying capacity of the axially compressed CFRP-strengthened CFS plain angle columns was presented in this paper. Plain angle specimens (nominal dimension—70 × 70 × 1.5 mm) were of 500 mm and 1000 mm in length. Both uni-directional (UD) and bi-directional (BD) carbon fiber fabrics were considered. Based on the obtained results of the considered specimens and various CFRP strengthening configurations, the following salient observations were made:

1. In the case of skin-strengthened specimens with a single-ply or layer of CFRP (i.e., SLSS), CF_UD-0° resulted in a peak increase in axial strength and stiffness and hence is recommended over CF_UD-90°, for which a marginal increase was observed.
2. In the case of skin-strengthened specimens with a double-ply or layer of CFRP (i.e., DLSS), CF_UD-0°/BD resulted in a greater increase in axial capacity and stiffness and hence is recommended over CF_BD/UD-0°.
3. No change in failure mode was observed due to CFRP skin-strengthening except that local buckling was minimized.
4. Apart from its contribution to the axial capacity of the composite section, the use of cardboard in-fill in addition to the CF_UD-0° wrapping transformed the open angle section into a closed solid section, which greatly enhanced the axial stiffness and strength. The failure was due to combined splitting of cardboard in-fill and the rupture of CFRP.
5. CFRP strengthening results in a significant increase in axial capacity and stiffness and hence is a viable lightweight strengthening that can be adopted in practice provided the initial cost is not a constraint.

## Data Availability

The datasets used and/or analyzed during the current study available from the corresponding author on reasonable request.
